# Internal Jugular Vein Thrombosis following Oropharyngeal Infection

**DOI:** 10.1155/2015/538439

**Published:** 2015-09-17

**Authors:** Asli Bostanci, Murat Turhan

**Affiliations:** Department of Otolaryngology-Head and Neck Surgery, Akdeniz University School of Medicine, 07070 Antalya, Turkey

## Abstract

Internal jugular vein thrombosis (IJVT) is a rare condition which may lead to life-threatening complications such as sepsis and pulmonary embolism. Prolonged central venous catheterization, intravenous (IV) drug use, trauma, and radiotherapy are the most frequent causes of the IJVT. IJVT that develops after the oropharyngeal infection is a quite rare situation today. In this paper, a 37-year-old woman was presented; swelling occurred on her neck after acute tonsillitis and she was diagnosed with IJVT through Doppler ultrasonography and magnetic resonance imaging and managed without complications. Early diagnosis and conservative treatment with broad-spectrum IV antibiotics and anticoagulant agents have a critical importance for the prevention of fatal complications.

## 1. Introduction

Internal jugular vein thrombosis (IJVT) is a rare vascular disease which may lead to life-threatening complications such as sepsis and pulmonary embolism [1]. Morbidity and mortality are high in the cases when correct and early diagnosis cannot be made. Its most frequent causes are the central venous catheterization and intravenous (IV) drug use [2]. Trauma, functional neck dissection, hypercoagulability, occult or known malign diseases, radiotherapy, and deep head-neck infections are the rare causes of the IJVT [3].

Contrary to the lower extremity deep vein thrombosis, data related to the clinical consequences of the IJVT are quite limited and differ depending on the underlying etiology. In this report, a case, which had IJVT development after acute tonsillitis, was presented in the light of literature.

## 2. Case Presentation

This study was conducted in accordance with the Declaration of Helsinki and with approval from the Ethics Committee of the Akdeniz University Hospital (Antalya, Turkey). Written informed consent was obtained from the patient.

A thirty-seven-year-old female patient admitted to our clinic with the complaint of a painless swelling on the left side of her neck, which had emerged one week before. It has been learnt that she was diagnosed with acute tonsillitis in another clinic, to which she applied with the complaints of fever, throat pain, and swallowing difficulty approximately two weeks ago and started to receive oral antibiotic treatment, but she did not apply the treatment regularly. In her history, she did not have any significant diseases, traumas or operations, IV drug use, or smoking story. On physical examination, a 3 × 2 × 1 cm in size, well-defined, semimobile, soft mass was detected along the front edge of the sternocleidomastoid muscle, on the left side of the neck. It had no hyperemia or heat increase on itself. No other pathology was detected in the ear-nose-throat examination; other system examinations were normal. In routine laboratory tests, hemoglobin was 9.6 g/dL, leucocyte 9090/mm^3^, thrombocyte 380000/mm^3^, sedimentation 120 mm/hour, prothrombin time 12.77 seconds, and partial thromboplastin time 33.0 seconds. Liver and kidney function tests were normal. The patient was hospitalized with the prediagnosis of deep neck infection and broad-spectrum IV antibiotherapy was initiated.

**Figure 1:**
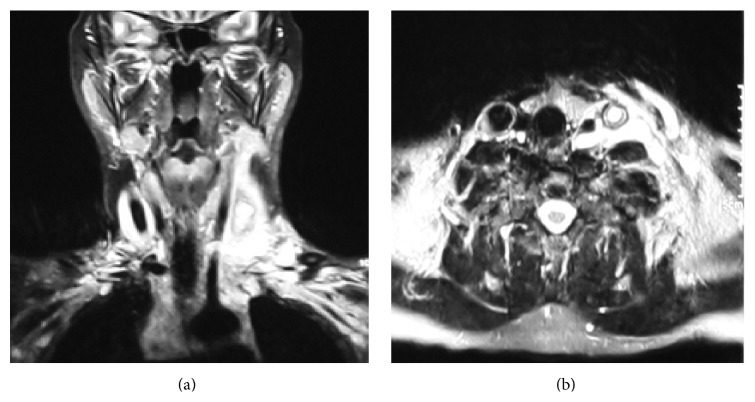
Magnetic resonance imaging of the left internal jugular vein thrombosis: (a) coronal plane, (b) axial plane.

No growth occurred in the throat culture. No pathology was detected in the chest radiography. While thrombosis was observed during the course of the left internal jugular vein in the Doppler ultrasonography (USG), no color filling was observed in the bilateral subclavian vein. However, venous drainage was detected at a low speed. Color filling and compression responses of the bilateral axillary veins were normal. During the magnetic resonance imaging (MRI), thrombosis-compliant appearance was observed in the left internal jugular vein and infection-compatible finding in muscle and fascia layers (Figure 1).

Low molecular weight heparin treatment was initiated for the case diagnosed with the IJVT. On the 7th day of the treatment, an explicit regression was observed in the symptoms of the patient and the thrombus was completely recanalized in the control Doppler USG. The patient, whom oral anticoagulant treatment began to be applied, was asymptomatically discharged. The patient is in the 6th month of her follow-up and no complications have been observed during this period.

## 3. Discussion

IJVT was firstly defined as a complication of the peritonsillar abscess [4]. It reflects the thrombus formation at any point during the course of the internal jugular vein. Venous thrombosis develops with the activation of the coagulation mechanisms that emerge secondarily for the normal blood flow failure. The physiopathology of thrombosis is explained with the factors related to endothelial injury, blood flow changes, and hypercoagulability which are known as “Virchow's triad” and frequently observed in the deep veins of the lower extremity [4].

Swelling and sensitivity are generally observed along the front edge of the sternocleidomastoid muscle during the physical examination of the IJVT cases [1–4]. Tovi et al. stated that a majority of the patients had fever (83%), leukocytosis (78%), swelling on throat (72%), and neck pain (66%), and pulmonary complication developed in 28% [3]. IJVT may be mistaken with deep neck infections, cellulite, painful lymphadenopathy, and head-neck tumors, which may cause painful swelling in the throat. However, as in our case, pain and heat increase may not appear all the time and no examination finding may be detected in terms of an infectious focus. Presence of a throat infection, IV drug use, or IV catheter story in the patient's history must cause suspicion and recall upper extremity deep vein thrombosis to mind.

Because contrast venography is an invasive technique besides being a gold standard in the IJVT diagnosis, it has many risks such as clot migration and septic embolism. Today, venography has been replaced by the Doppler USG, computed tomography, and MRI, which are noninvasive diagnostic methods [4]. Although USG is reliable, noninvasive, and cheap, it may be insufficient to evaluate the regions like skull base and mandible. MRI has advantages such as providing multiplanar images and nonexistence of radiation exposure.

The mechanism of the IJVT development following oropharyngeal infection is not exactly known. The general accepted thought is that oropharyngeal infection is directly spread from the parapharyngeal space towards the internal jugular vein through tonsils and fascial planes, or spread occurs through peritonsillar veins or lymphatics, and then, IJVT develops [5]. A clear differentiation between septic and nonseptic IJVT is the basis for clinical decision-making regarding antibiotic use, hospitalization of patients, and need for intensive care due to probable fatal complications. Lemierre's syndrome is the septic thrombophlebitis of the internal jugular vein. It is preceded by an oropharyngeal infection by anaerobic organisms and also known as postanginal sepsis. Clinical course of this syndrome is variable and complications may emerge depending on the involvement of any system [6].

In a retrospective case-control study including 23 patients with a septic thrombosis of the internal jugular vein, Schubert et al. [7] reported that fourteen patients needed intensive care unit treatment for a mean duration of 6 days and two of them developed severe acute respiratory distress syndrome. Anticoagulation therapy and IV antibiotherapy were given in 90% of patients, and all 23 patients survived the disseminated infection without consecutive systemic morbidity. Riffat et al. [8] stated in the case series, where they presented the 3 cases including IJVT development after tonsillitis, acute otitis media, and odontogenic sepsis, that IV antibiotherapy and therapeutic-dose anticoagulant treatment began to be applied to all the patients as per the culture result, venous recanalization was documented in all three cases after a 6-week treatment, and no complications were observed. Although our case did not have any clinical evidence of active infection, she was treated with broad-spectrum IV antibiotics combined with low molecular weight heparin due to recent history of oropharyngeal infection and risk of developing septic complications. On the other hand, in patients with nonseptic IJVT, treatment with anticoagulants alone may be appropriate.

In conclusion, observation of deep vein thrombosis in the upper extremity, axilla, and neck veins, contrary to what is observed in the lower extremity, is a rare situation and the data related to the clinical results of the patients in the group, in which IJVT developed especially following the head-neck infection, are quite limited. Early diagnosis and conservative treatment with broad-spectrum IV antibiotics and anticoagulant agents have a critical importance for the prevention of fatal complications.
